# Evaluation of the adverse events following immunizations surveillance system in Harare City, Zimbabwe, 2016: a descriptive cross sectional study

**DOI:** 10.11604/pamj.2017.28.308.12730

**Published:** 2017-12-13

**Authors:** Sithole Zvanaka, Juru Tsitsi, Prosper Chonzi, Gerald Shambira, Notion Tafara Gombe, Mufuta Tshimanga

**Affiliations:** 1 MPH Programme, Department of Community Medicine, University of Zimbabwe, Zimbabwe; 2 City Health Directorate, Harare, Zimbabwe

**Keywords:** Adverse events following immunization, surveillance, Harare City

## Abstract

**Introduction:**

Vaccines safety are monitored by looking for Adverse Events Following Immunizations (AEFIs). A review of the 2014 Harare City consolidated monthly return form (T5) revealed that 28 AEFIs were seen in 2014. However, only 21 were reported through the system. We therefore evaluated the Harare City AEFI surveillance system to assess its usefulness.

**Methods:**

A descriptive cross sectional study was conducted. Twenty one of 41 clinics were randomly selected and 51 health workers were randomly recruited. Interviewer administered questionnaires were used to collect data. Epi info 7 was used to generate frequencies, means and proportions.

**Results:**

Out of 51 respondents, 50 (98%) knew the purpose of AEFI system, 48 (94%) knew at least two presenting symptoms of AEFIs and 39 (77%) knew the correct date of form submission to the next level. Receiving no feedback 24 (47.1%), fear of victimisation 16 (31.4%) and work overload 11 (21.6%) were the major reasons for under reporting. Eighty six percent perceived the system to be simple and 43 (84%) were willing to continue participating. Fifty three percent (27) reported taking public health actions (such as awareness campaigns & making follow ups) basing on AEFI data collected. All 46 reviewed forms were completely filled and submitted in time. All 21 clinics had written AEFI guidelines and case definitions. Only 14 of 21 clinics had adequately stocked emergency drugs. The total cost for a single notification was estimated at US$22.30.

**Conclusion:**

The system was useful, simple, acceptable, timely, stable, representative but costly. The good performance of the system reported in this evaluation could be attributed to high health worker knowledge. Following this evaluation, replenishment of out of stock drugs and follow up of missing 2014 AEFI feedback from MCAZ were done. In addition, making the system electronic is recommended.

## Introduction

**Figure 1:**
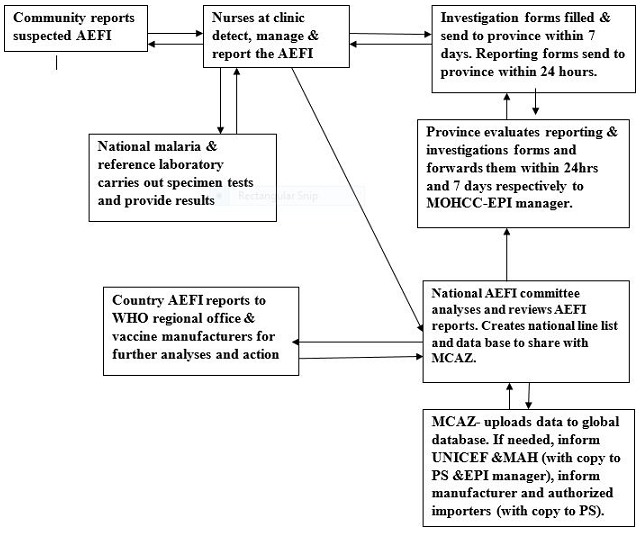
Flow diagram if the AEFI surveillance system, Harare, 2017

Public health surveillance is the ongoing, systematic collection, analysis, interpretation and timely dissemination of data regarding a health-related event for use in public health action to reduce morbidity and mortality and to improve health [1]. World Health Organization (WHO) defines Adverse Events Following Immunization (AEFI) as a medical incident that takes place after an immunization, causes concern and is believed to be caused by immunization [2]. These AEFI may be caused by a vaccine(s) or may occur coincidentally. They are classified into five categories which are vaccine reactions, program errors, coincidental events, injection reactions and unknown events [3, 4]. Globally, it is estimated that immunization averts an estimated two to three million deaths from diphtheria, tetanus, pertussis (whooping cough) and measles every year in all age groups [5]. The WHO in 1999, developed generic guidelines for AEFI surveillance that can be adapted to local resources and systems [6]. Zimbabwe was among the 66 countries that had been trained in AEFI surveillance by the WHO Global Training Network as of July 2004 [7]. The Expanded Program of Immunization (EPI) was launched in Zimbabwe in 1981 with the overall objective of reducing morbidity and mortality rates among children less than five years of age caused by vaccine preventable diseases such as tuberculosis (TB), diphtheria, whooping cough, tetanus, polio measles and Rubella [8]. Zimbabwe included the AEFI surveillance system in its EPI policy document by the year 2000 after realizing the world wide growing concern on safety of vaccines. The overall goal of the AEFI surveillance system is early detection and appropriate prompt response to adverse events in order to lessen negative impacts on immunization programs and the health of vaccines [9, 10]. The National Pharmacovigilance Centre, Medicines Control Authority of Zimbabwe (MCAZ) in collaboration with the Zimbabwe Expanded Program on Immunization are the main drivers of this initiative [11]. AEFI surveillance system contributes to the realization of Sustainable Development Goal (SDG) number three [12]. This is through achieving goal targets 3.2 (achieving universal health coverage through reduction of under-five mortality to at least 25 per 1000 live births.) and goal target 3.8 (achieving universal health coverage through access to safe vaccines). The ministry of health in Zimbabwe requires every adverse event to be notified [12]. Reporting is usually case-based as shown in Figure 1, however active surveillance based on search for selected medical events can be useful for specified events [13, 14]. Harare city has a system which is in line with this ministry's expectation. The T12 surveillance system for Harare city picked up 28 adverse events in 2014. However, only 21 out of 28 AEFIs were notified. In 2015, only 25 out of 28 were reported. The system is prone to under reporting. We therefore broadly set out to evaluate the adverse event surveillance system in Harare city, in 2016.

## Methods

A descriptive cross sectional study was carried out in Harare city. Fifty one health workers involved in AEFI surveillance were enrolled into the study. Twenty one of forty one clinics involved in AEFI surveillance were randomly selected. A minimum sample size of 50 was calculated. Forty two nurses found on duty were recruited into the study. All three district nursing officers and one EPI manager were purposively recruited as key informants. Five out of seven district medical officers were randomly selected as key informants. Completed AEFI notification forms from January 2014 up to December 2015 were reviewed to check for systems attributes like data quality, simplicity, completeness, sensitivity, predictive value positive and timeliness of the system. Interviewer-administered questionnaire were used to interview the health workers to determine their knowledge on the operations of the surveillance system and assess other system attributes. Data was captured and analyzed using Epi Info Version 7 to generate means, frequencies and graphs. Permission to carry out the study was sought from the institutional review board, Harare City and the Health Studies Office. Informed written consent was sought from all the interviewees and they were assured of confidentiality.

## Results

The study recruited 51 participants and of these 86.3% were females. The majority (82.4%) of the respondents were nurses. The median years in service of all participants was 8.5years (Q1 = 6; Q3 = 15) (Table 1).

**Table 1:** Demographic characteristics of health workers in Harare City, 2016

Characteristics	Frequency n (%) n = 51
**Gender**	
Female	44 (86.3)
Male	7 (13.7)
**Designation**	
District medical officers	5 (9.8)
District nursing officers	3 (5.9)
Nurses (RGNs and SIC)	42 (82.4)
EPI manager	1 (2)
**Median Years in Service**	8.5 (Q_1_ = 6Q_3_ = 15)

**Reasons for under reporting**: Reasons highlighted for under reporting of AEFI cases include receiving no feedback on AEFI (47.1%), fear of victimisation (31.4%), work overload (21.6%), too many data sources being required to fill the form (7.8%) and fear of exposing work incompetency (2%) (Table 2).

**Table 2:** Reasons for under reporting of AEFI surveillance System, Harare City, 2016

Reason	Frequency n (%) n=51
No feedback from MCAZ	24 (47.1)
Fear of victimization	16 (31.4)
Work overload	11 (21.6)
Too many data sources required to fill the form	4 (7.8)
Fear of exposing work incompetency	1 (2)

**Knowledge**: Out of the 51 respondents, 50 (98%) knew the purpose of AEFI system and were aware of who fills which form. Forty eight (94%) knew at least two presenting symptoms of AEFIs. Thirty nine (77%) knew the correct date of submission of form to the next level. Knowledge was assessed using 5 point Likert scale and rated as excellent, very good, good, fair and poor. The level of knowledge among nurses was deemed excellent (76%) (Table 3).

**Table 3:** Knowledge levels of health workers on AEFI surveillance system, Harare city

Knowledge on	Nurses n (%) n = 42	Doctors n (%) N = 5	DNOs & EPI manager n (%) N = 4	Total n (%) N = 51
Purpose of the system	41 (98)	5 (100)	4 (100)	50 (98)
Awareness of who fills the notification form	41 (98)	5 (100)	4 (100)	50 (98)
At least two presenting symptoms of AEFIs	39 (93)	5 (100)	4(100)	48 (94)
Correct date of submission to the next level	32 (76)	3(60)	4 (100)	39 (77)

**Simplicity**: Forty four of 51 perceived the system to be simple. Eleven of 51 cited the need for the system to be further simplified since it requires too many data sources when it comes to filling of the forms. Out of the 42 nurses, only 28(67%) had ever identified an AEFI and completed reporting forms. Twenty-five out of the 28 (89%) respondents reported taking less than 30 minutes to fill the forms. with the majority of them mentioning an average of 25 minutes.

**Acceptability**: The majority of the nurses 41 (98%) of 42 felt it was their duty to fill the AEFI notification forms. Thirty four (83%) of 42 nurses and 9 (100%) of nine key informants were willing to continue participating in the system. The 46 forms identified had all areas completely filled. All adverse events were reported to the districts within 24hours. Minutes for the three meetings held and two audits conducted in 2016 were available hence the system was deemed acceptable.

**Stability**: All 21 clinics (100%) reported using cell phones to notify the District Nursing Officers (DNO) and the EPI manager. All sites (100%) had AEFI notification forms at the clinic. All 21 clinics had written guidelines. Thirty nine nurses (93%) stated that they had written guidelines and out of them only 32 (82%) nurses had these guidelines readily available. All 21 clinics (100%) had AEFI case definitions, of these 16 (76%) were displayed on the wall. Only 14 out of 21 clinics (67) had adequately stocked emergency drugs in case of an AEFI emergency. All clinics were manned by AEFI trained nurses.

**Representativeness**: The system was representative. All the 21 health facilities participated in AEFI surveillance.

**Flexibility**: Of the 51 respondents 32 had ever filled the notification forms, all DNOs reported to ever fill the investigation portion of the notification forms. None of the DMOs reported having filled the notification forms. However, they all reported that the forms are flexible since they have space for additional information to be added. The additional space was seen.

**Predictive value positive (PVP)**: Of the 21 and 25 notification forms reported to MCAZ in 2014 and 2015 respectively, only 25 forms from 2015 were investigated and had their results sent back to Harare City as feedback from MCAZ. Twenty three out of 25 notified AEFIs were confirmed vaccine related. Therefore the calculated system's PVP for the cases reported in 2015 was 92%. There was no feedback on all AEFIs notified in 2014 hence we could not calculate the PVP for 2014.

**Timeliness**: The 46 adverse events reported through the AEFI surveillance system had all forms completely filled and were reported to district and province within 24 hours. Therefore the system was timely.

**Data quality**: All 46 AEFI forms had all sections completely filled and reported on time. The quality of data was excellent. The calculated systems' PVP for the 2015 forms was 92%.

**Usefulness**: All the 51 respondents (100%) alluded to the fact the AEFI surveillance system is useful and reported that AEFI data is used at local level. However 27(53%) respondents reported to have taken public actions based on AEFI data which included awareness campaigns (8%), intensifying the health education campaigns 67%), making follow ups (17%) and stopping student nurses on attachment from administering vaccines (8%). Thirty five respondents (86%) reported that an audit on AEFI was being done. Minutes for the audit were availed. However 24 (47.1%) of 51 respondents stated that they were not getting feedback on AEFI investigation results from MCAZ at the time of study. Official communication on part of the feedbacks given was verified and confirmed. We concluded that the system is useful since it influenced public health actions taken locally (Table 4).

**Table 4:** Usefulness of AEFI Surveillance System, Harare city, Zimbabwe, 2016

Usefulness	N (%), n=51
Any public health actions	27 (53)
Any feedback given	27 (53)
Any audits done	44 (84)
Achievement of systems objective	50 (98)

**Cost of running the system**: The total estimated cost for a single notification to Harare city headquarters was US$22.30. assuming a salary of US$1200 per month for health worker completing the form, 22 working days per month, 8 hour working day, the cost of filling a form for 30 minutes = US$3.40 per adverse event. The investigation process was also estimated to be US$3.40. Assuming an average distance of 15km between the clinics and the head office, the estimated cost for transporting forms $10.50 for a “to and from ” journey. The cost of stationery and airtime for a single notification was estimated to be US$ 4.40.

## Discussion

The high levels of health workers' knowledge on AEFI surveillance system in Harare City might have been influenced by the existence of experienced staff (median years of service 8.5) among health workers. This is not consistent with findings by Pfute et al, 2006, on a similar study in Bubi district, where they found out that the system was affected by lack of health workers' knowledge [15]. The differences in knowledge levels in these studies might be due to differences in the staff experience. Bubi district staff (median years in service -1.75). This therefore shows that health workers knowledge on a particular surveillance system has a great influence on its success. While every effort is made to ensure that vaccines used in national programs are safe and effective, it has been shown that adverse reactions do occur in some people [16]. Reporting, investigation, correct management, prevention and transmission of correct information to the public on all adverse events is important to ensure the sustainable implementation of the EPI program. AEFI surveillance system in Harare city is prone to under reporting as some of T5 reported adverse events were not investigated. Studies have shown the tendency of under reporting in all passive AEFI surveillance systems and this is type of surveillance in Zimbabwe as well as Harare city [17]. The health workers reported work overload, fear of victimization by superiors, lack of feedback and complexity of the form to fill as reasons for under reporting. This gives the impression that even if the staff were going to detect an adverse event, that event was not going to be reported by some of the respondents thereby increasing the chances of under reporting. Key informants' interviews revealed that six out of nine key informants confirmed the existence of nurses' vacant posts in their districts.

This threatens the stability of the system. WHO 2013 stated that other studies on AEFISS reported not knowing the reporting process, inability to find forms, fear that reports will lead to personal consequences, guilt about having caused harm and being responsible for the event as barriers to reporting [18]. The surveillance system was found to be stable since most of the resources for the system were available at health facilities. However internet services in clinics were not yet functional at the time of the study. This threatens the stability of the system as this may result in delays in transmission of data to higher levels. From the study findings nurses were short staffed but well experienced and were willing to run the system effectively. Stability was further aided by the availability of stationery. There was low level of emergency preparedness in cases of emergency in Harare city clinics. This is consistent with Ncube et al (2005) findings on a similar study in a similar setting (city of Bulawayo), who found out that there were low levels of emergency preparedness among Bulawayo city health clinics [19]. The system was reported by most health workers to be useful. However, a minority of the participants had ever received feedback on AEFI surveillance. These findings indicate that though the majority of health workers reported the system to be useful, its usefulness is not evidenced by reported feedbacks. This poses a threat to usefulness of the system and could be improved if MCAZ gives timeous and religious feedback on AEFIs reported. Taking “action” is what distinguishes a surveillance system from merely data recording [18]. Dembedza et al 2008, in their study on assessment of the AEFI surveillance system in Zaka district also showed that apart from health workers reporting the system as useful no data was captured at the clinics on AEFIs and no public health action was taken at local level based on AEFI surveillance data [20]. However, with Harare city public health actions are being taken.

## Conclusion

We therefore concluded that this system is useful, simple, acceptable, flexible, timely, stable but costly. HCW were highly knowledgeable. Possible reasons for under reporting were fear victimisation by the superiors, work overload and lack of feedback from MCAZ on AEFIs. Emergency trays were not adequately stocked. Strengthening of replenishment of emergency trays, making system electronic and reporting of feedback by MCAZ were recommended.

### What is known about this topic

- All events that are actively notified to the health care system by the parents/guardians or patients themselves or identified by a health care provider are supposed to be submitted to the MCAZ where assessment for causality according to the causality assessment of an AEFI.

### What this study adds

- This study shows how some of the AEFI surveillance systems function in other different settings. It highlights some of the challenges faced in the system implementation.

## Competing interests

The authors declare no competing interests.
